# Mechanisms of breast cancer metastasis

**DOI:** 10.1007/s10585-021-10090-2

**Published:** 2021-05-05

**Authors:** S. David Nathanson, Michael Detmar, Timothy P. Padera, Lucy R. Yates, Danny R. Welch, Thomas C. Beadnell, Adam D. Scheid, Emma D. Wrenn, Kevin Cheung

**Affiliations:** 1 Department of Surgery, Henry Ford Cancer Institute, 2799 W Grand Boulevard, Detroit, MI, USA; 2 Institute of Pharmaceutical Sciences, Swiss Federal Institute of Technology, Zurich, Switzerland; 3 Department of Radiation Oncology, Massachusetts General Hospital, Harvard Medical School, Boston, MA, USA; 4 Wellcome Sanger Institute, Cambridge, UK; 5 Department of Cancer Biology, University of Kansas Medical Center and University of Kansas Cancer Center, Kansas City, KS, USA; 6 Translational Research Program, Public Health Sciences and Human Biology Divisions, Fred Hutchinson Cancer Research Center, Seattle, WA, USA; 7 Molecular and Cellular Biology Graduate Program, University of Washington, Seattle, WA, USA

**Keywords:** Genes, Breast cancer evolution, Mitochondrial DNA, Cell clusters, Lymph node and systemic metastasis

## Abstract

Invasive breast cancer tends to metastasize to lymph nodes and systemic sites. The management of metastasis has evolved by focusing on controlling the growth of the disease in the breast/chest wall, and at metastatic sites, initially by surgery alone, then by a combination of surgery with radiation, and later by adding systemic treatments in the form of chemotherapy, hormone manipulation, targeted therapy, immunotherapy and other treatments aimed at inhibiting the proliferation of cancer cells. It would be valuable for us to know how breast cancer metastasizes; such knowledge would likely encourage the development of therapies that focus on mechanisms of metastasis and might even allow us to avoid toxic therapies that are currently used for this disease. For example, if we had a drug that targeted a gene that is critical for metastasis, we might even be able to cure a vast majority of patients with breast cancer. By bringing together scientists with expertise in molecular aspects of breast cancer metastasis, and those with expertise in the mechanical aspects of metastasis, this paper probes interesting aspects of the metastasis cascade, further enlightening us in our efforts to improve the outcome from breast cancer treatments.

## Introduction

### S. David Nathanson and Michael Detmar

The scientifically based management of breast cancer (BC) is dependent upon an understanding of the natural history of the disease. Initially only direct treatment of the diseased breast was possible, and surgery played a predominant role well into the middle of the twentieth century [1]. Treatment by radical mastectomy was offered as an ‘all or nothing’ response; the practitioner could do a drastic operation with the hope that all the cancer had been removed. Patients and their families to this day ask the surgeon whether he/she ‘got it all.’ Until fairly recently that also meant removing all the lymph nodes in the axilla, even when the tumor had not spread to those nodes, exposing the patient to an uncomfortable series of postoperative complications, including severe lymphedema of the arm.

The procedures and processes that played out in the imagination of physicians treating patients with BC were based upon a mechanical/anatomic understanding of metastasis. In a sense, everything in the metastatic process could be related to ‘tubes,’ namely, blood vessels and lymphatics, and their connections in the breast, axilla, and the systemic circulation; tumor cells traveled through these vessels to other parts of the body where they might invade the end organ, such as the lung, liver, bone or brain, forming metastases. Once vital organs had succumbed to these invasive tumors the patient would eventually die of the disease.

When radiation [2] was added to the armamentarium of treatment for BC, it was initially only used for locoregional management on the superficial chest, axilla and supraclavicular areas, but it later became useful as well for direct treatment of metastases in distant organs. The mechanisms of metastasis were still not explored beyond the understanding of an anatomic component; the ‘maps’ of metastasis were known to involve lymphatics, blood vessels, and end points, much like geographic maps that indicate roads, rivers, cities, mountains, coast lines and states.

Systemic treatment for BC, in the form of chemotherapy and endocrine manipulation, advanced the management of BC and was based upon a developing understanding of tumor cell biology, particularly the mechanisms by which tumor cells proliferated [3]. Proliferation did not explain why tumor cells metastasized, although cells that landed in other organs proliferated just like they had in the primary site in the breast. These systemic treatments were often directed at symptoms, such as bone pain.

Evolution and revolution in the management of BC has focused on multidisciplinary treatment and most of the advances have been directed at decreasing metastasis by decreasing proliferation; cells that do not proliferate seem not to metastasize. Many of the large clinical studies of the past few decades [4] have focused attention on preventing metastasis by treating the patient with adjuvant therapies, potentially killing microscopic metastases because that also improves survival. These advances have been accomplished knowing where but not how BC metastasizes.

We have begun a new phase in the systemic treatment of BC by focusing on molecular targets that can be attacked, such as the well-known HER-2/neu, which is based upon molecular aberrations in the tumor cells, but not proven to be involved in how tumors metastasize. Some molecular targets could be functionally important in how tumor cells metastasize without necessarily affecting their ability to proliferate. Drugs targeting functionally important molecules could potentially stop tumors from metastasizing and result in prolonged survival of the patients.

Studies in animal models and in vitro at the dawn of the experimental metastasis era [5] showed the importance of tumor cell invasion into surrounding tissues, with adjusted biological processes already known in cell biology, such as chemotaxis, proteolytic enzyme secretion, expression and de-expression of adhesion molecules, the development of new blood and lymphatic vessels in and around tumors, and immune reactivity [6]. Early pharmacologic studies, using drugs that target biochemical stages in metastasis, show some promise but the field is in its infancy.

The guidelines for BC management, based upon high quality clinical studies have become very dependent upon molecular markers and on statistical models at predicting metastasis [7]. For example, the Oncotype DX test looks at 16 genes, some of which may be functionally important in metastasis, to predict which estrogen-receptor positive tumors that have not metastasized to regional lymph nodes (RLNs) are likely to metastasize to systemic sites [7]. Clinicians frequently use this and other gene studies to decide which patients might benefit from chemotherapy.

The Henry Ford Cancer Institute Mini Symposium on the mechanisms of BC metastasis was held in October 2019 in San Francisco as part of the 8th International Cancer Metastasis Congress. The main objectives of this mini symposium were to look at some recent studies on how tumor cells behave and travel to systemic sites, either through the RLN or directly into the systemic circulation at the site of the original tumor. The more we know about how BC metastasizes at a microscopic, cellular and molecular level, the more likely we are to develop new, more effective ways of treating BC.

## The potential role of the sentinel node in systemic metastasis

### Timothy P. Padera

The presence of lymph node metastasis (LNM) is associated with worse clinical outcomes for cancer patients than those without LNM [8–10]. The question becomes: “Why is this true?” Is it because LNM is just a biomarker for the aggressiveness of the primary cancer, in which more aggressive tumors spread to distant sites and drive patient mortality [1, 11]? Or is it that LNMs themselves can drive cancer progression by serving as a source for distant metastases [12–15]?

There is new urgency to address these questions as 4 randomized clinical trials (three breast cancer studies-ASCOG-Z0011, IBCSG 23-01, and AMAROS; one melanoma study MSLT-II) have shown that additional lymph node resection in patients beyond the sentinel lymph node does not provide any survival benefit when adjuvant radiation therapy and systemic therapies are used [16–20]. However, implicit in this strategy is the potential to leave cancer bearing lymph nodes in the patient that will require further therapy. Radiation therapy of RLNs has been shown to improve outcomes (disease-free and cancer-specific survival) in early-stage BC [21, 22]. Thus, treatment of lymph nodes benefits patients, highlighting the importance of LNMs in driving cancer progression.

To begin to understand how LNMs could be driving cancer progression, we asked whether it was possible for cancer cells that have colonized lymph nodes to escape the lymph node and seed distant metastatic sites. The main challenge in addressing this question is the ability to identify cancer cells that had been in a lymph node and then were later either circulating in the blood or residing in a distant metastatic site. To overcome this challenge, we relied on a photoconvertible fluorescent protein, Dendra2, which when exposed to specific wavelengths of ultraviolet light can change the color of its emission from green to red [23]. After stably expressing Dendra2 in 3 murine cancer cell lines (4T1 triple negative breast carcinoma, B16F10 melanoma and SCCVII squamous cell carcinoma), each tumor type was grown orthotopically in syngeneic immunocompetent mice [24]. Once the cancer cells spontaneously metastasized from the primary tumor to the lymph node, the primary tumors were surgically resected. We then exposed only the tumor draining lymph node to the photoconverting light and were able to switch the color of the cancer cells in the lymph node from green to red with an efficiency of about 70%. After this photoconversion, the only source of red cancer cells in the animal could be from LNM. We then asked, can we identify red cancer cells from the lymph node circulating in the blood or in distant metastatic sites?

First, we collected all the blood from the animals and using flow cytometry, identified red cancer cells—which must have been in the lymph node at the time of photoconversion—as well as green cancer cells in the blood of animals containing photoconverted 4T1 and B16F10 LNMs, but identified only green cancer cells in the blood of animals containing photoconverted SCVII LNMs [24]. These data provided the first direct evidence that in some models, metastatic cancer cells in lymph nodes can escape the node and enter the blood circulation. Next, we collected the lungs from these animals, made sections through the whole lung and used confocal microscopy to spectrally scan through the tissue. Similar to the blood, we identified red cancer cells, as well as green cancer cells, in the lungs of animals containing photoconverted 4T1 and B16F10 LNMs, but identified only green cancer cells in the lungs of animals containing photoconverted SCVII LNMs [24]. These data provide evidence that the circulating cancer cells that escape the lymph node can disseminate to a distant metastatic organ.

To determine if cancer cells can also escape directly from the primary tumor, we photoconverted a portion of 4T1 primary tumors before they spread to the lymph node and identified circulating red cancer cells in the blood [24]. Thus, there are two sources of circulating cancer cells: those directly from the primary tumor and those from the LNM. Further experiments showed that cancer cells both directly from the primary tumor and those from spontaneous LNM can form metastatic lesions in the lungs [24].

The next question we addressed was how cancer cells escaped the lymph node. There are two possible exit routes. First, cancer cells could leave via the efferent lymphatic vessels, heading to the next echelon nodes upstream. Clinically, it is observed that LNM can often be found in secondary draining lymph node beds and cancer cells have been observed in medullary lymphatic structures in metastatic lymph nodes from extramammary Paget’s disease [25], making escape through the efferent lymphatic vessels plausible. However, by performing intravital microscopy of metastatic lymph nodes, we saw cancer cells migrating toward and interacting with lymph node blood vessels [24]. We established that cancer cells in our mouse model migrate and enter lymph node blood vessels, an additional route of escape for cancer cells out of the lymph node. Finally, looking at a series of LNM from patients with head and neck squamous cell carcinomas, we identified cancer cells inside lymph node blood vessels in 7 out of 19 cases [24], confirming that cancer cells in some patients are able to escape the lymph node through nodal blood vessels.

Our data show it is possible for cancer cells to spontaneously disseminate to lymph nodes and then escape the lymph node to seed another metastatic site. The work also provides evidence that one method of escape from the lymph node is by invasion of the lymph node blood vessels by the cancer cells. Our work does not exclude other methods of dissemination to distant metastasis (e.g., direct invasion of primary tumor blood vessels, which we show is also occurring in the 4T1 model) or other methods of escape from the lymph node (e.g., escape through efferent lymphatic vessels). It also does not suggest that every patient will have distant dissemination occurring from metastatic lymph nodes. However, our data did provide direct evidence for a new route for how cancer cells can spread throughout the body, one that had long been hypothesized [13, 26]. Our work was simultaneously corroborated by data from the laboratories of Dontscho Kerjaschki and Michael Sixt, which showed similar findings using different methods [27].

Other recent work studying LNM in patients also supports the concept that cancer cells can metastasize from lymph node lesions. In a study of 17 colorectal cancer patients, Naxerova et al. built phylogenetic trees to relate the primary tumor, LNMs and distant metastases based on mutational evolution. In this study 6 out 17 patients showed LNM with close mutational relationships to distant metastases [28]. Similarly to our mouse data, this study also shows that some, but not all, patients may have distant metastases seeded by lymph node lesions. In a long-term study of 3329 BC patients that underwent RLN biopsy, Nathanson et al., show that LNMs are predictive of distant metastases, whereas lymphovascular invasion at the primary site is not predictive by itself [29]. These data further suggest that LNM can drive distant cancer progression.

Our work has shown that it is possible for LNM to spread further in the body. However, there is currently no actionable information to identify which patients are at risk for this occurring. Further research into the molecular and physiological mechanisms that drive this process are needed before at-risk patients can be identified and interventions designed. By identifying these mechanisms, we aim to improve outcomes for patients with LNM while balancing the risk of overtreatment by appropriate patient selection.

## The genomic evolution of breast cancer metastasis

### Lucy Yates

Cancer is primarily caused by DNA damage. The transformation from a normal to cancerous phenotype and from primary to metastatic BC is marked by the accumulation of genetic changes known as somatic mutations. By applying evolutionary principles to genomic sequence data, we have started to uncover the fundamental patterns underlying BC metastasis.

Whole genome sequencing studies have revealed that BC genomes are highly abnormal. In a study of 560 primary BCs, we identified an average of 6214 single base pair substitutions, 665 small insertions and deletions and 140 structural variants per cancer [30]. In a separate study, we found that the progression from primary tumor to the diagnosis of distant metastatic disease was accompanied by an increase in the mutation burden of around a third [31]. Only a tiny fraction of the somatic mutations detected in cancer genomes alter known cancer genes and act as drivers of cancer progression. The majority of mutations are ‘passengers’ with no effect cell fitness; however, they provide great statistical power for identifying mutational signatures and tracing the evolutionary patterns that underlie cancer metastasis.

#### Cancer subclones

Breast cancers are composed of multiple genetically related subclones [32–37].

Subclone composition can vary over space (within the same tumor mass or across metastatic sites) and time (sequential samples). All subclones in a cancer are by definition genetically related, having arisen from a single common ancestor, but individual subclones are distinguished by the existence of private genetic changes. This may be directly observed in single cell studies although technical limitations still mean that these are most reliable for measuring copy number variation rather than point mutations [34, 38]. Subclonal composition can also be inferred indirectly from bulk tissue samples (that typically consist of thousands of millions of mixed cancer cells and normal cells) using bioinformatic approaches that determine the nature of and cellular prevalence of genotypes using features such as variant allele fraction, tumor purity and allele specific copy number information [32, 39]. Multi-region sampling approaches increase our ability to detect subclonal structure. Recent advances in low-input material sequencing (of 100 cells or less) are moving this approach to a new level, providing much higher resolution of subclone composition in both cancer and normal tissues. In the coming years, we expect these approaches to continue to provide important insights into how normal tissue development is subverted during cancer evolution [40, 41].

Different genetic subclones can have different phenotypes and therefore provide the substrate upon which natural selection may act. Indeed, we and others have demonstrated that aggressive cancer traits such as invasion, treatment resistance or metastasis can be mapped back to antecedent subclones earlier in the disease history [31, 32, 41–43]. Understanding how these ill-fated subclones relate and differ to their seemingly well-behaved sisters, within the same primary tumor, is a major priority in cancer research. The recent development of a wide range of spatial sequencing approaches is set to provide critical insights into the spatial arrangement and characterization of individual cancer subclones [44–46]. Combining these techniques with evolutionary mapping approaches offers an exciting opportunity to isolate and study the most clinically important subclones within the tumor environment.

#### Trees describe cancer evolution

**Fig. 1:**
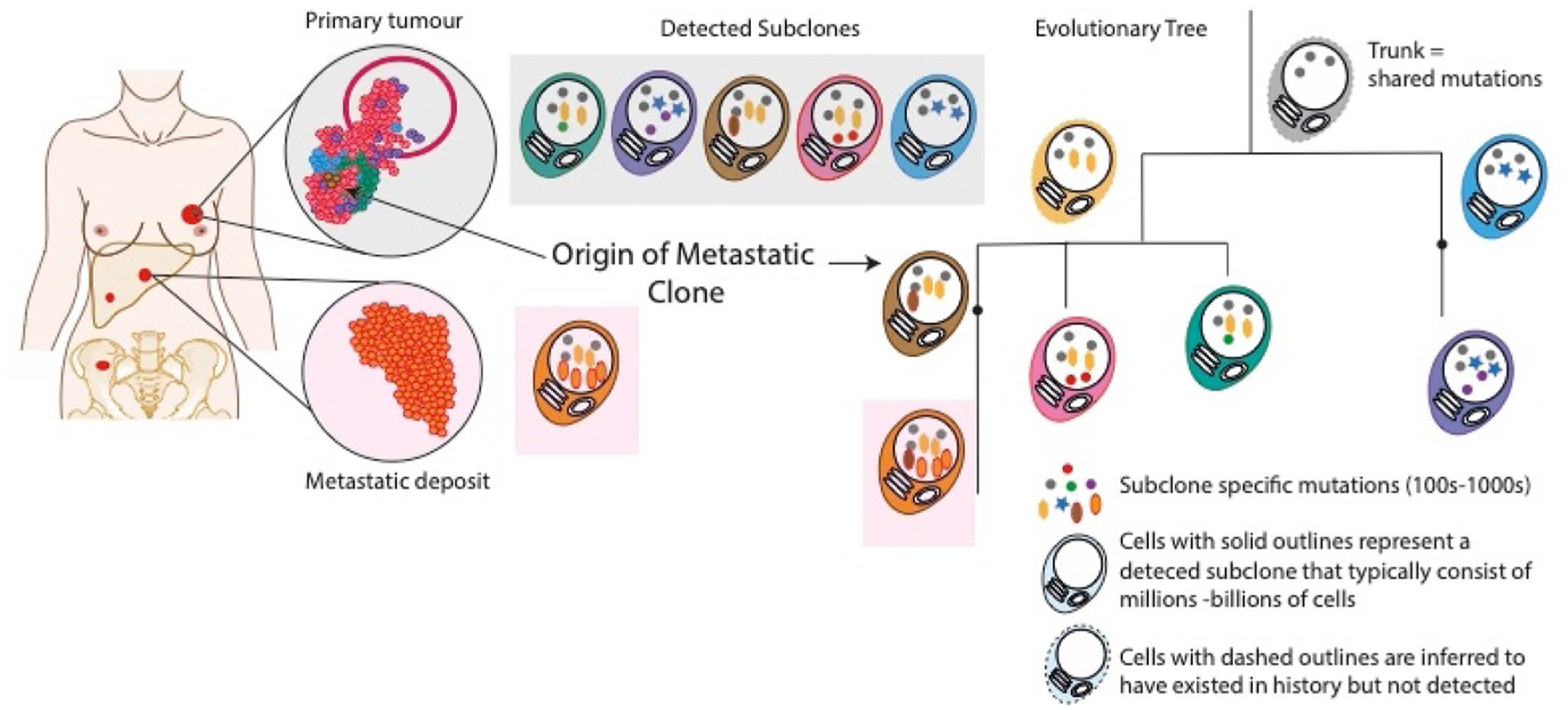
Evolutionary or phylogenetic trees provide a map of cancer development and progression. Cancer subclones are represented by different colored cells with distinct mutation combinations

We can use evolutionary or phylogenetic trees, akin to the ‘tree of life’ famously described by Charles Darwin, to represent diagrammatically, the inferred evolutionary relationships between different cancer subclones (Fig. 1). Phylogenetic tree construction starts with two basic assumptions: mutations can be gained but not lost and each mutation can only occur once [47]. Although there are caveats to these assumptions, these can generally be accommodated. Firstly, we must consider copy number differences between subclones (i.e., where deletions might have ‘lost’ some mutations in one subclone/sample) and secondly, by using as much of the rich genome-wide mutation data as possible rather than relying on limited targeted gene panels that only identify a handful of mutations at best. Using the two principles we can identify ‘clonal’ mutations as those shared by all cancer cells and form the ‘trunk’ of the phylogenenetic tree (Fig. 1). Mutual exclusivity of mutations identifies subclones from different branches, while co-occurrence indicates mutations within the same subclone or a direct lineage. The lengths of branches are typically scaled according to the number of mutations, and therefore, act as a kind of molecular clock that allows us to order branching events in time. Studying the branching patterns that we derive can then help us to understand how different cancers develop and progress.

#### Metastasis seeding cell dissemination timing

The point at which the Metastasis Seeding Cell (MSC) diverges form the primary tumor has important implications for the use of adjuvant systemic therapy. Take the example where 3 driver mutations have accumulated within a primary tumor up to the point where it is diagnosed. A precision medicine treatment plan would have to be based on the 3 mutations seen in the primary tumor, but we do not know how representative this is of the potential MSCs at this time point. In the situation where the MSC left the primary tumor recently, i.e., the late branching scenario, it is likely that these cells share the same 3 driver mutations and the primary tumor can be considered a good proxy. However, in the case where the MSC departed from the tumor a long time ago, i.e., an early branching scenario, these cells are less likely to contain the same driver mutations seen in the primary tumor and furthermore, they have had significant time to accumulate novel driver mutations that we will not be able to detect. The primary tumor in this situation is a poor proxy, providing inaccurate and incomplete information about the cancer cells that we wish to eliminate.

When we applied this principle to 17 cases of metastatic or relapsed BCs, we found that most MSCs diverged from the primary tumor late in evolution (on average 87% in primary tumor molecular time). This was a phenomenon shared across distant metastases, local relapses and synchronous LNM. Importantly, the majority of driver mutations in the primary tumor were also present in 100% of the cells in the metastasis indicating that targeted therapies based on the primary tumor genomic profile would be relevant to the metastasis.

Some studies appear to contradict our findings, suggesting that cancer cell dissemination occurs, or at least can occur early—even from pre-invasive lesions [48–52]. Notwithstanding the need to extend our analyses to larger sample series and different BC subtypes, we do suspect that one potential confounding factor is that these studies have tended to rely on less robust technologies for inferring clonal relatedness. Indeed, much of the support for early dissemination is derived from disseminated tumor cell (DTC) studies that defined bone marrow cells as DTCs based on morphology and cytokeratin expression. Importantly, recent single cell sequencing of DTCs and exome sequencing/genome-wide copy number profiling of the primary tumor revealed that only a fraction of presumed DTCs are actually clonally related to the primary tumor [53]. Further work linking these DTCs to the subsequent metastasis is needed to determine if real-time characterization could be helpful for treatment scheduling during the relapse free window.

#### Future perspectives

Our findings to date have identified that metastases typically arise from a detectable subclone in the primary tumor. To date, we have not been able to demonstrate that the driver mutations that dominate the metastasis are pre-selected in the primary tumor. Furthermore, we have had little success in identifying the anatomical localization of the subclones that are most closely related to a future metastasis. A critical question remains to be answered: “Is there something special about the metastasis seeding subclone?”.

The emergence of spatially resolved approaches, such as laser capture microscopy with low-throughput sequencing and in-situ sequencing of somatic mutations, are expected to allow much closer interrogation of primary tumors and pin-pointing of ill-fated subclones [41, 44]. These approaches will be essential for identifying subclone specific associations with tumor micro-environmental factors that are likely to be important determinants of subclone fate.

## Mitochondrial-nuclear exchange (MNX) mice reveal contributions of mitochondrial genetics to cancer metastasis

### Thomas C. Beadnell, Adam D. Scheid, and Danny R. Welch

#### Introduction

The ability of cancer cells to metastasize requires coordinated expression of multiple genes [54–64] and the efficiency of metastasis is contributed to by multiple quantitative trait loci (QTL) [65] alleles that influence a trait [66] in combination with environmental factors.

Because of its comparative size, most studies with QTL examine the nuclear genome, ignoring the mitochondrial genome. However, in the context of cancer, mitochondria are key players, which were first identified by Warburg with regard to their roles in metabolism [67–69], but the definitive mechanisms by which mitochondrial DNA (mtDNA) contribute to cancer phenotypes has been understudied and underappreciated. It is critical to emphasize that mtDNA polymorphisms are likely metastasis *modifiers* rather than drivers per se. mtDNA QTL would combine nuclear and mitochondrially-encoded genes to regulate cancer severity and metastasis [70–72]. Given the miniscule size of the mitochondrial genome (~ 16 kb) to the nuclear genome (~ 3 × 10^4^ kb), could the hypothetical existence of mtDNA metastasis QTL be reasonable? Despite being relatively understudied, the answer is that it is likely (reviewed in [73–75]).

The classic experiment demonstrating the existence of metastasis QTL in the nuclear genome was performed in the laboratory of Kent Hunter, who crossed female mice from multiple *Mus musculus* strains to male transgenic mice with the oncogenic polyomavirus middle T antigen (PyMT) under the control of the mouse mammary tumor virus (MMTV) promoter (FVB/N-TgN(MMTV-PyMT)) [76]. Critically, these mice were made on the FVB/N genetic background [77]. Crossing inbred strains with FVB/N-TgN(MMTV-PyMT) resulted in differential tumor latency and metastatic burden in the first filial generation [76]. Using backcrosses and comprehensive genetic screens, his group has identified metastasis modifiers [78–80], many of which have also been observed in human BCs [81–83].

However, an alternative interpretation of their data was recognized because their experimental design crossed females of those various strains to male FVB/N-TgN(MMTV-PyMT) mice. Since mtDNA is maternally transmitted, the possibility existed that mtDNA could also be a metastasis modifier QTL. A growing volume of data implicates mitochondria in cancer and metastasis (reviewed in [73, 74]). Specifically, Kaori Ishikawa and colleagues demonstrated that transfer of mitochondria from highly metastatic cells to poorly metastatic recipient cancer cells enhanced metastatic potential. A reciprocal experiment also lowered metastatic efficiency [84].

We began exploring approaches to study contributions of mtDNA to metastasis. Unfortunately, the unique characteristics of mtDNA presented numerous experimental challenges (reviewed in [73, 85]). Briefly, mtDNA is present in 100s to 1000s of copies per cell, making alteration of *all* mtDNA copies currently impossible even with the most advanced methods [86]. Therefore, introduction of heterogeneous heteroplasmy would confound effects of the alteration. The use of cybrids or transmitochondrial mice involves prior exposure to mutagens, which could potentially introduce mutations that would complicate interpretation. Likewise, generation of conplastic mice, while not exposing to mutagenic agents, involves backcrossing for at least 10 generations to achieve 99.9% nuclear DNA (nDNA) purity with different mtDNA composition [87]. While conplastic mice mostly eliminate disparate nDNA as a confounding variable, the number of necessary backcrosses increases the likelihood that nDNA recombination has occurred.

To circumvent the issues associated with the above approaches, we developed the mitochondrial-nuclear exchange (MNX) mouse model [88] to assess whether there are metastasis modifier QTLs in the mitochondrial genome. Crossing MNX female mice with mammary cancer transgenic mouse models demonstrated a driver-dependent regulation of metastasis. Moreover, injection of syngeneic cancer cells into MNX mice revealed non-cell autonomous contributions of the mitochondrial genome to metastasis. mtDNA polymorphisms alter immune profiles as well as other changes to the tumor microenvironment, which affect metastatic efficiency. Together, these data demonstrate that mtDNA indeed contains QTL for cancer metastasis.

Generating MNX mice did not involve backcrossing or mutagen exposure. They were made using micropipette transfer of pronuclei from embryos into enucleated cytoplasts from another mouse strain (i.e., having mitochondria from another strain) [88, 89]. The resulting hybrid embryos transplanted into pseudopregnant mice resulted in progeny, which are homoplasmic, phenotypically normal and fertile. The resulting colonies have been maintained for more than a decade and have been very stable.

#### Mitochondrial DNA alters metastasis in a cell autonomous manner

Female MNX mice on the FVB genetic background having FVB mitochondria (FF), BALB/c mitochondria (FB) or C57BL/6 mitochondria (FC) were crossed with MMTV-PyMT mice. The nuclear genomes were identical except for the transgene. Latency to first tumor formation and metastasis were measured. FF crosses developed tumors as expected while time to first tumor was accelerated in the FB crosses and slowed in FC progeny. Almost identical to the data from the Hunter crosses, pulmonary metastatic burden (i.e., cumulative volume of metastases and metastasis size) differed. The number of metastases arising was relatively unaffected [90].

Subsequently, the same MNX mouse strains were crossed to male FVB/N-Tg(MMTVneu) mice, which overexpress the wild-type Her2/neu oncogene under the control of the MMTV promoter [91]. Tumor latency and metastases were quantified. Results in FF and FC progeny were similar to those seen in the PyMT model. However, Her2 FB mice developed tumors later and had lower metastatic burden than FF mice [91]. This contrasted with the PyMT crosses, where FB mice exhibited more rapid primary tumor formation than FF mice.

Taken together, these two studies demonstrate that mtDNA polymorphisms affect both tumorigenicity and metastasis. The changes are dependent upon contributions of both the nuclear genome and the mitochondrial genome, exactly what one would expect for QTL. Additionally, Brinker and colleagues showed that using male MNX mice crossed to MMTVneu females (i.e., mtDNA from the MNX mice would not be inherited in the progeny) resulted in no difference in the tumor behaviors.

Additional evidence that the mitochondrial genome could impact tumor formation was obtained by Vivian et al. [92]. Mice unexposed to carcinogen treatments were allowed to age naturally, and the incidence and location of spontaneous autochthonous tumors were recorded. Although underpowered for some strains of MNX mice, a tumor protective effect of C57BL/6 mtDNA was observed.

Since metastasis involves multiple genes to be coordinately expressed and only a very small fraction of those genes are encoded in the mtDNA, we reasoned that SNP in mtDNA could influence gene expression in the nuclear genome. By altering the nuclear epigenome via changes in cytosine methylation or modifying histones, gene expression and corresponding phenotypes could be affected. We performed whole genome methylation sequencing coupled with RNA sequencing and determined that there were selective changes in the location of methylation in the mouse genome and that some of those changes occurred concomitant to changes in gene expression [93]. In a more recent study, we measured 4 common histone marks using ChIP-Seq and again found a selectivity in location of histone modifications. Many of the methylation marks and histone marks are in similar regions, suggesting that mtDNA somehow dictates how the nuclear genome operates.

#### Mitochondrial DNA alters metastasis in a non-cell autonomous manner

Although having previously demonstrated that nuclear [87] and mitochondrial [64, 69] genetics could alter metastatic efficiency, it was recognized that all cells in F_1_ progeny would inherit mtDNA from the MNX mother. We therefore investigated the role of mtDNA in the tumor microenvironment and its impact on tumor development. Using 2 mammary carcinoma and 2 melanoma cell lines, we were able to keep the mtDNA constant within the cancer cells and injected the cells into either WT or MNX mice. Orthotopic tumor growth was not significantly altered; however, formation of experimental metastasis was significantly affected. These experiments utilized wild-type C57BL/6 (CC), C3H/HeN (HH) and reciprocal MNX mice C57BL/6 nDNA:C3H/HeN mtDNA (CH) and C3H/HeN nDNA:C57BL/6 mtDNA (HC) into which cells were injected either orthotopically or into the lateral tail vein. Critically, wild-type mice and their nuclear-matched MNX counterparts were major histo-compatibility matched; so, overt transplant rejection mechanisms were eliminated as an experimental variable. Clearly the mtDNA in stromal compartments influenced behavior of tumor cells [94]. The question is: how?

In all mouse strains utilized for the above studies, only a handful of mtDNA polymorphisms have been identified [71]. Changes have been reported in protein encoding genes as well as in transfer RNA [71, 92]. Polymorphisms in mtDNA genes encoding electron transport chain proteins implicate metabolism and previous studies found oxygen consumption to be different between MNX strain mammary tissue [69] and cardiomyocytes [68]. However, parameters associated with metabolism (i.e., oxygen consumption, extracellular acidification, and mitochondrial load) showed no correlation with changes in tumor cell behavior. Metabolomic analyses comparing the MNX mice also identified metabolite differences among MNX strains. Nothing jumped out as an explanation for the different tumor cell phenotypes observed. The caveat to dismissing differences in metabolism as a mechanism for stromal manipulation of metastasis is that the measurements were done in vitro and in tissues that are not necessarily the most appropriate locations of tumor cells.

A byproduct of electron transport is generation of reactive oxygen species (ROS). We tested the hypothesis that altered ROS in MNX mice might be responsible for the changes in metastatic potential. Using MitoTEMPO, a scavenger for mitochondria-derived ROS, the metastasis-promoting effects of C3H/HeN mtDNA were reduced. While the results are consistent with the hypothesis, reciprocal experiments (i.e., increasing ROS in C57 mice) could not be done because of multiple off-target effects [91].

A key stromal contributor to tumorigenicity and metastasis is the immune system. Based upon linkages between mitochondrial genetics, metabolism, and observations that metabolism is closely linked to immune differentiation and polarization [95], we studied whether baseline immune profiles and functionalities could explain results in the non-cell autonomous studies. Most differences between mouse strain immune profiles were found to be regulated by nDNA. Interestingly, many of the lymphocyte populations were not dramatically altered. However, macrophage differentiation (and perhaps polarization state) was significantly altered by SNP in the mtDNA [96]. Detailed studies are underway to refine the changes and to directly test which macrophage subpopulations are most relevant to the phenotypic changes reported for tumorigenicity and metastasis.

#### Relevance and perspective

The co-evolution of mtDNA and nDNA [97] demonstrate clear communication between the two genomes. Metabolic adaptations to changing climates encountered by ancient humans migrating from Africa provided selective pressures dictating divergence from the original mtDNA genotype contained in ‘Mitochondrial Eve’ [98]. Those physiological adaptations mediated by mtDNA haplogroups have played critical roles in human evolution. Moreover, relevant to this discussion, they also contribute to disease pathologies and racial health disparities [73, 99]. Individuals with certain mtDNA haplogroups have increased predisposition to certain cancers compared to people with other mtDNA haplogroups [85, 90, 91]. Some adaptive advantages in some mtDNA variants resemble oncogenic mitochondrial functions [100, 101].

Clinicians have long known that patient race/ethnicity contributes to cancer incidence rates and survival. For instance, triple negative BC is more prevalent in African American and Hispanic Caucasian women [102, 103] who exhibit higher proliferation rates, increased angiogenesis markers, higher grade, higher rates of LNM and worse overall survival compared to non-Hispanic women [104–113]. While economic and modifiable factors contribute to outcome disparities, incidence and progression differences persist even after controlling for socioeconomic factors [106, 109–113]. To date, no nuclear genomic explanations have been found comparing patients from different races [114–117]. We posit that the impact of contributions from each QTL that define race are masked by uncontrollable variables in analyses. Use of the MNX model, which isolates mtDNA as an experimental variable, allowed us to gain a foothold into the mechanistic underpinnings of mitochondrial contributions to metastasis.

In the context of metastasis, it is critical to remember that tumor cells encounter multiple different microenvironments throughout their journey from the primary tumor to distant sites. Our data (Table 1) exploring the roles of mtDNA in the MNX mice support the notion that mitochondria perform critical functions in the receipt of information as well as the conveyance of neoplastic cell signals to the microenvironment. While the mitochondrial genome is small, it leverages the much larger nuclear genome to affect neoplastic cell behavior. Mitochondria are mediators of outside-in and inside-out cellular communication [118–120]. They sense changes in the microenvironment and signal to the nucleus the requirement of a cell to adapt. As yet, the mediators of mitochondrial signaling are poorly defined. Identifying those signals represents an as yet untapped opportunity for prognostic information from the mitochondria and therapeutic targets within the mitochondrial genome. There is currently no direct evidence that mitochondrial genetics play a role in human breast cancer. Efforts to create Synteny maps between mouse mitochondrial and human mitochondrial genomes and a comprehensive map of mtDNA polymorphisms are underway.

## Deconstructing collective metastasis: emergent multicellular mechanisms supporting metastatic colonization

### Emma D. Wrenn and Kevin J. Cheung

A large number of tumor cells reaching distant sites will die and never grow into a clinically detectable metastasis [121]. Disseminated tumor cells encounter inhospitable stromal matrices, cell types, and paracrine signals different from their organ of origin. In addition, they are actively eliminated by immune cells, such as natural killer (NK) and T-cells. Diverse mechanisms have been described that enable tumor cells to overcome these barriers: entry into a stem cell-like state, epithelial-to-mesenchymal and mesenchymal-to-epithelial transitions, genetic mutation, and co-option of the native microenvironment for example [122–124]. Here, we focus on an emerging mechanism by which cancer cells increase their chance of success—metastasizing as cohesive clusters of cells, also known as collective metastasis.

Much research has been conducted on the mechanisms by which single tumor cells metastasize. However, accumulating studies have shown that tumor cells can also metastasize as multicellular aggregates, and do so with much higher efficiency than solitary cancer cells [125, 126]. A key foundation for this concept originates from seminal experimental metastasis studies performed in the mid-twentieth century. Investigators injected lung cancer, melanoma, and fibrosarcoma tumor cells into mice as either clusters or filtrated single cells [127–129]. In each instance the clusters had significantly greater success forming new metastases. Since then, a number of studies in different tumor types and metastasis models have demonstrated greater metastasis formation by injected clusters compared to single cells [130–133], with for example ~ 15-fold higher lung metastasis seeding rates by clusters in pancreatic cancer [134] and up to 500-fold higher rates in BC [135, 136] compared with equal numbers of single cells.

These findings are buttressed by recent studies tracing the clonal composition of spontaneous metastases in mouse models. Multiple groups have used multi-color fluorescent tumor cell models [131, 132, 134, 135, 137–139] or deep sequencing [140, 141] to demonstrate that transplantable primary tumors produce metastases that are polyclonal, that is seeded by multiple clones present in the primary tumor. A large fraction of metastases were found to be polyclonal in several of these models; 48–53% using BC cell lines [137], 54% using BC PDXs [131], ~ 80% of large lesions to the peritoneal wall and diaphragm using KPCX pancreatic cancer cells [134], and over 97% using the MMTV-PyMT breast tumor model [135]. A caveat to these experiments is that polyclonal metastases could arise from serial seeding by single cells [142]. Importantly, multiple studies have also conducted experiments excluding large contributions from serial seeding, which indicates that polyclonal metastases in their models arise primarily from tumor cell clusters [132, 134, 135, 137].

Deep sequencing studies comparing primary and metastatic genomic alterations have also supported a role for polyclonal seeding in human tumors. Polyclonal seeding has been reported in colorectal cancer [143–146], intrahepatic cholangiocarcinoma [147], gastric cancer [148], and prostate cancer [149]. Two recent studies of metastatic BC patients identified polyclonal metastases in 63% to 73% of patients [150, 151]. This clonal diversity raises the possibility that multiple subclones in polyclonal metastases could cooperate to increase one another’s fitness, instead of responding to selective pressures from the tumor microenvironment purely on their cell-intrinsic properties. This kind of interclonal cooperativity has been observed in mouse models of cancer [139, 152, 153]. But further research is needed to understand how diverse genetic compositions in tumor cell clusters contribute to different stages of collective metastasis. Importantly, direct isolation of tumor cell clusters (CTCs) from the blood of metastatic patients has provided further clinical evidence for collective metastasis [154–157]. Moreover, CTC clusters are correlated with poorer patient prognosis across the most common cancer types [137, 158–167]. Together these diverse experimental and clinical studies indicate that tumor cell clusters are potent metastatic seeds.

Despite these many studies establishing the impact of cluster-based metastasis, the molecular mechanisms underpinning their efficient colonization are much less understood. One possible explanation is physical entrapment; it could be that clusters’ large size simply results in rapid arrest in the circulation. However, a recent study demonstrated that patient-derived CTC clusters in the blood could rearrange into single-file chains, which survive passage through narrow capillaries [168]. Additionally, another study in zebrafish models found no significant difference in the rate of extravasation between single and clustered CTCs [130]. These findings do not rule out the importance of physical entrapment. But, as discussed below, recent studies also show that clustering of tumor cells alters their properties beyond mechanical trapping in ways that support metastasis.

**Fig. 2:**
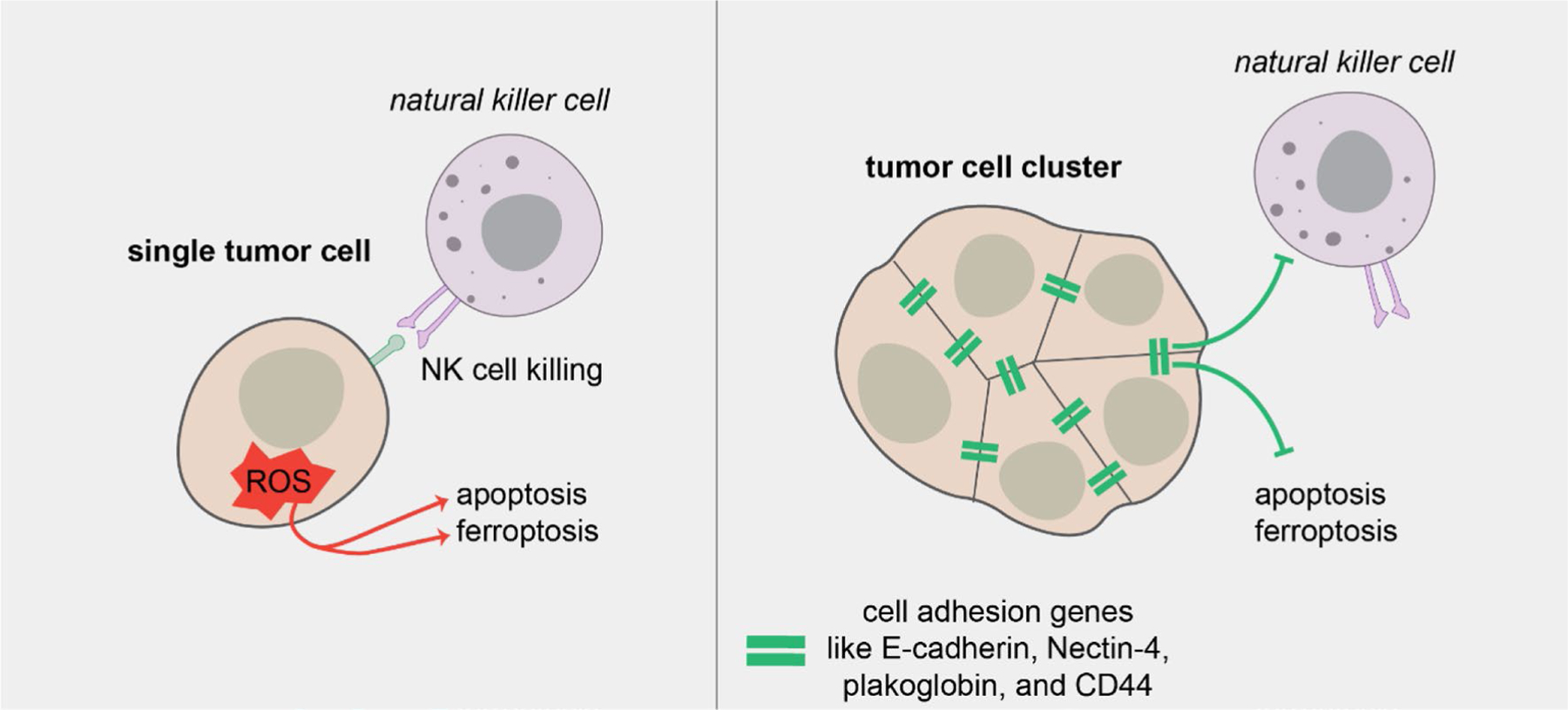
Clusters resist programmed cell death. Tumor cell clusters have increased survival at metastatic sites through several mechanisms including depletion of reactive oxygen species, resistance to NK cell killing, and pro-survival signals transduced downstream of cell–cell adhesion

The metastatic potential of a single cell can be conceived as a balance of factors promoting or hindering that cell’s ability to colonize other tissues. But to understand cluster-based metastasis we have to consider an additional dimension, the interactions amongst the cells in that cluster. Indeed, a number of recent studies have coalesced around a common theme: tumor cell clustering induces global shifts in key cell states promoting survival, growth, and ultimately colonization. For instance, clusters may be able to resist attrition in the critical early stage of colonization (Fig. 2). Shortly after tail vein injection, tumor cell clusters arrested in the lungs had lower rates of apoptosis than single BC cells [137]. Recently, we observed that while both clusters and single cells were able to reach the lungs immediately after tail vein injection, clusters persisted while single cells were mostly cleared within 48 h [136]. Additionally, time lapse imaging in the presence of an apoptosis biosensor revealed that clusters generated from 8 of 10 human BC tumor samples were significantly more apoptosis resistant than single cells in 3-dimensional culture [136]. A recent clinical study of CTCs from small-cell lung cancer patients also observed a reduction in apoptosis in CTC clusters vs. single cells [159]. Another study using BC tumor cell clusters from patient-derived xenografts found that homophilic CD44 adhesions promote cluster-based metastasis, possibly through PAK2-mediated increases in cell survival [131]. Together, these suggest that likelihood of survival during colonization is a key difference between clusters and single tumor cells.

Some of the survival advantage of clusters appears to depend upon resisting ROS, a critical challenge for disseminating tumor cells [169, 170]. A recent study found that E-cadherin mitigates TGFb-dependent ROS stress in tumor cell clusters, facilitating their survival at metastatic sites [171]. Clusters have also been shown to induce mitophagy to limit ROS and increase cell survival [172]. Clustering may play a role in non-apoptotic forms of cell death as well. Recent reports show that multicellular aggregates are more resistant to ferroptosis [173, 174], an iron-dependent form of non-apoptotic cell death characterized by ROS accumulation [175]. Tumor cell clusters generated Nectin-4 dependent Src signaling, which buffered against ferroptotic lipid peroxidation [173, 174].

Cell–cell adhesion could also help tumor cell clusters successfully evade attack from the immune system. Tumor cell clusters were recently demonstrated to be more resistant to NK cells than single cells via downregulation of NK cell activating ligands [132]. NK cells play a key role in the immunosurveillance and targeting of metastasis, and NK tumor cell infiltration and activation often correlate with better patient prognosis [176–178]. Interestingly, a number of cell–cell adhesion molecules function as NK cell inhibitory ligands, suggesting that NK cells may more effectively target solitary tumor cells or cells that have undergone complete epithelial mesenchymal transition EMT [132, 179, 180]. Clusters may even utilize the immune system to promote survival and growth; heterotypic clustering of CTCs with neutrophils has been found to promote cell cycle entry in tumor cells [181] and clusters may shift NK cells into a more metastasis-promoting state [182].

Beyond survival, clustering generates changes in signaling and cell state supportive of metastatic outgrowth. Tumor cell clusters are more proliferative and more stem cell-like than single tumor cells [131, 135, 136, 183]. How does cell–cell contact generate these pro-metastatic states? An attractive hypothesis is that cell–cell adhesion molecules directly generate pro-proliferative signaling through cross-talk with cellular signaling pathways that regulate growth [184]. On the other hand, many studies have shown that cell–cell adhesion induces contact inhibition, restricting cell proliferation [185, 186]. In fact, the same cell–cell adhesion molecule can either promote or inhibit metastasis depending on context. For example, E-cadherin is required for successful metastasis in a mouse model of BC and promotes cell survival [171]. But in the same mouse model, E-cadherin activating antibodies inhibit metastasis, suggesting that E-cadherin’s role in metastasis may be tunable [187]. At present, the balance between pro- and anti-metastatic signals downstream of cell–cell adhesion molecules remains unresolved and may be highly context-dependent between different cell types, tumor types, and model systems.

**Fig. 3:**
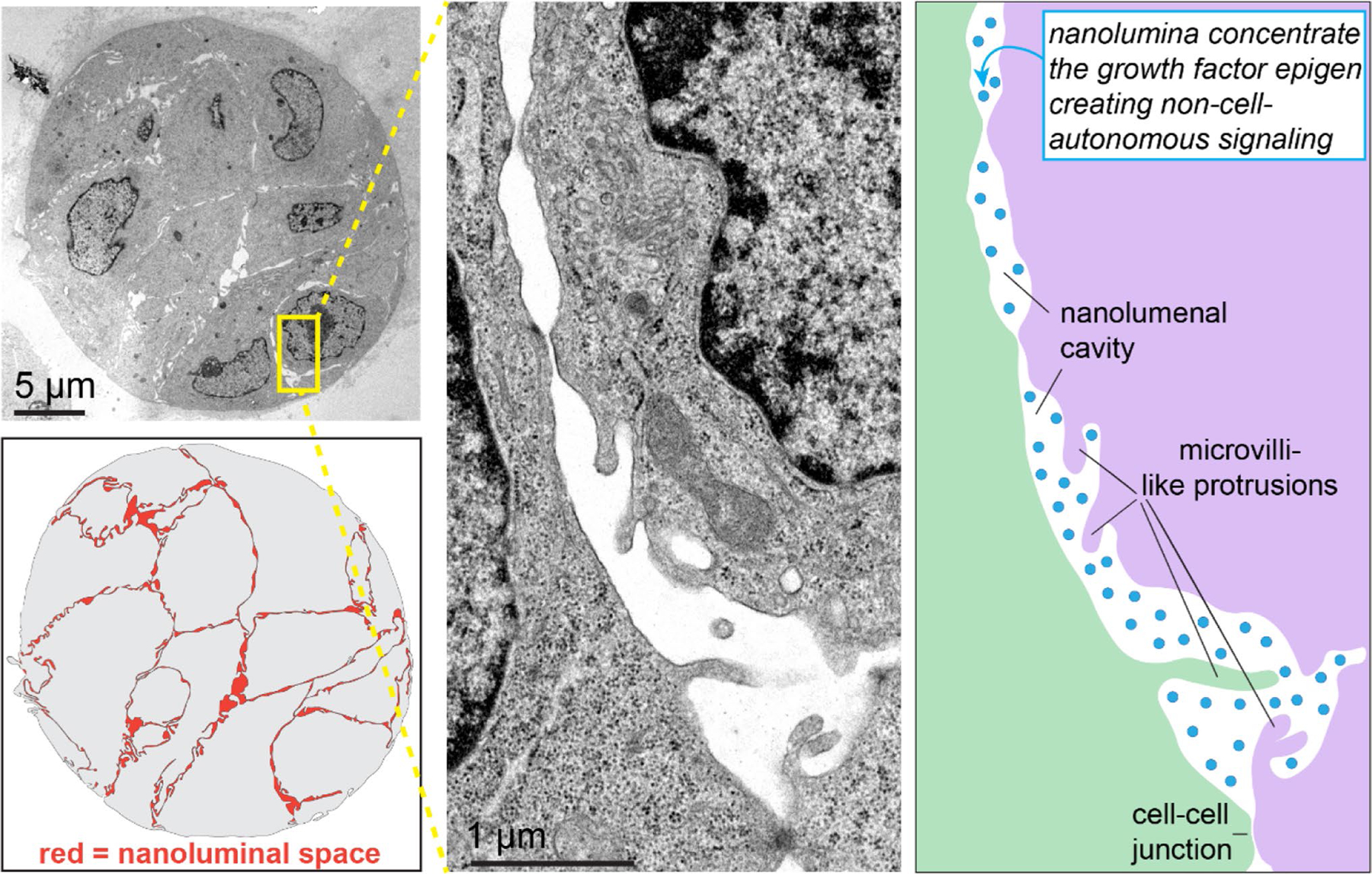
Tumor cell clusters contain intercellular nanolumina that concentrate signaling molecules. Left, transmission electron microscopy of an MMTV-PyMT tumor cell cluster. Between tumor cells we observe intercellular cavities lined by microvilli-like protrusions and sealed by cell–cell junctions. We find that the growth factor epigen is trafficked to and concentrated within nanolumina, resulting in cooperative pro-growth signaling during collective metastasis

Cell–cell adhesions can also promote metastasis without direct intracellular signal transduction. Instead, clustering can induce 3-dimensional architectural changes that generate collective signaling. Our group recently characterized nanolumina within tumor cell clusters (Fig. 3)—hollow spaces between cells, sealed at either end by electron dense cell–cell junctions [136]. They are often lined by microvilli-like protrusions, which can interdigitate between neighboring cells and provide high surface area for intercellular interactions. Nanolumenal junctions were selectively permeable, thereby controlling the composition of nanolumina and shielding them from the microenvironment.

Importantly, we found that nanolumina formed sites for intercellular communication. We identified a growth factor, epigen, which was trafficked into nanolumina where it achieved concentrations > 5000-fold higher than those outside the cluster. Importantly, epigen suppression profoundly reduced both primary tumor growth and metastatic outgrowth. By secreting a growth signal into a shared space, clusters create collective, non-cell autonomous signaling to promote proliferation during metastasis. In this instance, cell–cell adhesions do not directly transduce a signal but instead regulate a signaling molecule’s concentration by trapping it within intercellular cavities. This 3-dimensional topology-dependent collective signaling is an emergent feature of clusters, as it is not possible in single cells lacking cell–cell adhesion. Our findings indicate that the physical architecture of a multicellular cluster provides an additional layer of regulation during metastasis, and a mechanism for robust intercellular signaling.

Importantly, we also observed nanolumina in freshly isolated human tumor cell cluster samples. Nanolumina with restricted permeability were also present in an aggressive subset of basal-like 2 (BL2) triple negative BC cell lines which highly express epigen, but not in mesenchymal-like triple negative BC cell clusters. BL2 BCs have poor treatment response and overexpress growth factors and myoepithelial genes, whereas mesenchymal-like BCs overexpress genes related to EMT and cell motility [188–191]. RNA-sequencing analysis demonstrated that BL2 nanolumina-containing cell lines highly express epithelial genes and genes associated with branching morphogenesis during early development. This suggests that epigen expression and nanolumina may be linked to epithelial identity and could have a role during epithelial development that is exploited by metastatic tumor cell clusters.

Treatments are critically needed to effectively eradicate metastases and suppress the metastatic process [192]. An understanding of the mechanisms underlying tumor cell clusters’ metastatic potency could pave the way toward therapies targeting their metastatic advantages. One approach is to target the adhesion molecules holding clusters together. Indeed, disrupting cell–cell adhesion can repress metastatic potential [131, 137, 171, 183, 193]. However, cell–cell adhesion molecules are often highly expressed in normal tissues, which could narrow the therapeutic window unless tumor specific properties or activation states are identified. Another approach is to target the cooperative signals generated by clusters within nanolumina. However, much remains to be learned about the optimal strategy to target these collective signaling compartments in practice. Our finding that BL2 but not mesenchymal-like BCNot cells contain nanolumina also suggests that the relative contribution and mechanisms of collective metastasis could differ widely between cancers, subtypes, and individual patients. Future studies are essential to expand on these mechanistic insights into collective metastasis, to rigorously assess their relevance to specific subgroups of patients, and ultimately to translate these findings into the clinic.

### Conclusions

Breast cancer metastasis is a complex process requiring many interacting components, anatomic, cellular and molecular/genetic. Mechanical factors play a part in sentinel lymph node metastasis, and breast cancer cells may gain direct access to the systemic circulation by invasion into veins in the node. Polyclonal multicellular tumor cell aggregates metastasize with much higher efficiency than solitary cancer cells, partly because large size clusters might simply arrest in the circulation by physical entrapment. DNA studies show that some breast cancers metastasize to systemic sites without first entering lymph nodes, suggesting the secretion of gene-induced functional proteins, cytokines and/or peptides, present in some clones but not others, cause direct invasion into blood vessels in the primary breast tumor. It is more likely that tumor cells invade lymphatic rather than blood vessel capillaries at the primary site.

Identification of activated genes and other molecular markers that are important in systemic breast cancer metastasis would be valuable to clinicians in many ways. For example, a more accurate molecular fingerprint of tumor cells identified by needle biopsy of the primary tumor could subclassify patients into those who might not benefit from sentinel node biopsy. Innovation and adaptation will be necessary for continued relevance of SLN biopsy, which will likely take place in several areas. There would need to be incremental advances helping to optimize an already good technique. Other avenues of research could advance SLN utility even further, including developing more refined criteria for selecting patients for SLN biopsy. Some advances may make SLN biopsy redundant; for example, we can imagine non-surgical therapies that will kill tumor in lymph nodes in which case removing them might become unnecessary. We still don’t know for certain whether removing the SLN treats breast cancer and that will require further research.

In our current practice most patients with no clinical evidence of axillary lymph node metastasis who undergo lumpectomy for invasive disease are eligible for sentinel lymph node biopsy. Only one in four patients in this cohort are found to have node metastasis. This means that 75 of every 100 patients with negative lymph nodes might safely avoid the operation of sentinel node biopsy altogether. Revealing currently unknown molecular markers might help us identify these patients upfront. Seven in every seventy-five patients without node metastases develop systemic metastases. More accurate and advanced molecular signatures might allow us to focus adjuvant systemic therapy on only this small proportion of patients, which would be a major advance on our current use of commercially available molecular subtypes used to decide which patients should be offered chemo, targeted and hormonal therapies. The majority of patients could perhaps safely avoid uncomfortable systemic and loco-regional treatments without jeopardizing their chances of survival.

The development of anti-metastatic therapies is an obvious future direction for researchers who identify metastasis-related molecular markers. Hypothesis-directed research would first need to connect the clinically important molecular markers to the mechanisms of metastasis. For example, although we know that patients with HER-2/neu positive breast cancers are at higher risk for systemic metastasis, there is no direct proof that HER-2/neu is involved in the process of metastasis. Similarly, triple negative breast cancer carries a risk of poorer survival than hormone-receptor rich tumors, but no-one has yet identified any related molecular or cellular mechanisms to account for this difference. The relative contributions and mechanisms of breast cancer metastasis might vary widely between cancers, subtypes, and individual patients and we are at the dawn of those discoveries.

The precise biochemical and molecular/genetic signals that prompt lymphatic or blood vessel invasion, and methods by which tumor cells survive the hazardous journey through the blood stream, and how exactly those cells settle into their new environment within the body, are still under investigation but it seems likely that when we discover more details about these mechanisms we will be better able to target steps in metastasis formation. When we reach those goals we may be able to eliminate metastases altogether, goals that are both urgent and possible and will augur a new era of breast cancer treatment.

## Figures and Tables

**Table 1:** MNX mice and corresponding mtDNA-directed phenotypes

MNX mouse abbreviation	nDNA composition	mtDNA composition	Tumor latency	Metastasis size	No. metastases	nDNA methylation
FB	FVB/NJ	BALB/cJ	PyMT [90]:↓ Her2 [91]:↑	PyMT:↑ Her2:↑	PyMT: NS Her2:↓	Yes [93]
FC	FVB/NJ	C57BL/6J	PyMT:↑ Her2:↑	PyMT:↓ Her2:↑	PyMT: NS Her2:↓	Yes
CH	C57BL/6J	C3H/HeN	ND	ND	↑B16-F10 and K1735-M2 experimental metastasis	Yes
HC	C3H/HeN	C57BL/6J	ND	ND	↓B16-F10 and K1735-M2 experimental metastasis	Yes

MNX, mitochondrial-nuclear exchange; ND, not done; NS, not significant

Phenotypes are relative to wild-type strains with matching nDNA
